# Coping and family functionality in women with diabetes and breast cancer

**DOI:** 10.3332/ecancer.2026.2088

**Published:** 2026-03-12

**Authors:** María Valeria Jiménez-Báez, Sofía Concepción Thomas-Gómez, Gabriel González-Guerrero, David Rojano-Mejía, Eduardo Patricio Achurra-Godinez

**Affiliations:** 1 Family Medicine, Instituto Mexicano del Seguro Social, Coordinación de Planeación y Enlace Institucional, Hospital General Regional 17, Cancún, Quintana Roo 77533, México; 2 Family Medicine, Instituto Mexicano del Seguro Social, Unidad de medicina familiar 16, Coordinación de educación e investigacion en salud, Cancún, Quintana Roo 77533, México; 3 Social Service Intern, Instituto Mexicano del Seguro Social, Unidad de medicina familiar 19, Coordinación de educación e investigacion en salud, Cancún, Quintana Roo 77518, México; 4 Physical and Rehabilitation Medicine, Instituto Mexicano del Seguro Social, Coordinación de investigacion en salud, Ciudad de México 01210, México; 5 Social Service Intern, Instituto Mexicano del Seguro Social, Hospital General Regional 17, Coordinación de educación e investigacion en salud, Cancún, Quintana Roo 77533, México; a https://orcid.org/0000-0002-9114-4741; b https://orcid.org/0009-0002-2084-6612; c https://orcid.org/0009-0007-2256-3356; d https://orcid.org/0000-0002-6340-8463; e https://orcid.org/0000-0002-3700-5687

**Keywords:** breast neoplasms, family relations, diabetes mellitus, type 2, coping skills, clinical evolution

## Abstract

**Introduction:**

Breast cancer (BC) is the second leading cause of death in Mexican women. Comorbidity with type 2 Diabetes Mellitus (T2DM) is a risk factor for complications.

**Objective:**

To evaluate the evolution of family functionality and coping in patients with BC and T2DM after 4 years of diagnosis.

**Method:**

Longitudinal cohort study in women with BC and T2DM in Quintana Roo. Admission at the time of confirmed diagnosis of BC, discharge at 6 years of evolution. We studied: family function, coping, clinical, family and metabolic control variables.

**Results:**

65 women, of whom 18 concluded the study. Family functionality (FF-SIL) 51.1 versus 44.72. Coping level: Problem solving 4.66% (0,10.8) versus 6.2% (5.6,44.4); Self-criticism 24.6% (14.9,33.8) versus 4.6% (0,34.9); Emotional expression 21.5% (11,32.3) versus 4.6% (0,38.9); Desiderative thinking 26.2% (14.9,37. 4) versus 1.5% (0.18.3); Social support 4.6% (0.11) versus 1.5% (0.18.3); Cognitive restructuring 3.1% (0.9.7) versus 4.6% (0.38. 9); Problem avoidance 6.2% (1.5,13.8) versus 3.1% (0,27.8); Social withdrawal 9.2% (1.5,20.4) versus 1.5% (0,22.2) *p* = >0.5.

**Conclusion:**

The level of coping worsens over 4 years of evolution. Patients maintain their autonomy in decision making within the family. At diagnosis, families adapt; however, with the evolution, they infer sequelae of family health.

## Introduction

Breast cancer (BC) is the most diagnosed malignant neoplasm in the world. Most cases occur in women and represent a leading cause of premature death among women living in developing countries. First, in 2020, 2.2 million cases of BC were reported and found that 1 in 12 women will face this disease at some point in her life. Of the total reported cases, 685,000 resulted in death.

Second, in Mexico, it is the most common malignant neoplasm in women. Over the last three decades, its incidence and mortality have increased; a trend associated with changes in lifestyle and the ageing of the affected Mexican population. It has become the second leading cause of death among women in Mexico.

Furthermore, the American Diabetes Association in 2021, defines diabetes as the progressive loss of B cell secretion, resulting in insulin resistance and the worldwide rate is 8,812% in >20 years old population.

In 2019, Mexico reported a rate of 13.5%, a significant difference compared to global figures. In 2022, ENSANUT (National Health and Nutrition Survey) reported a rate of 18.3% (15.27 million cases), making it the second leading cause of death among Mexican women.

The risk of BC associated with type 2 diabetes mellitus (T2DM) was evaluated and the results were variable. An Argentine study from 2019 demonstrated that having T2DM leads to a higher risk of developing BC in stage II (14%), stage III (21%) and stage IV (16%) BC, compared to stage I. BC presents higher mortality in diabetic patients, specifically in women with a long history of the disease and cardiovascular conditions.

A descriptive epidemiological study in Mexico, conducted in a Family Medicine Unit found that there was a weak relationship between 71 patients with both BC and T2DM.

BC has one of the highest rates of impact on family structures. The World Health Organisation defines family as ‘a social group organised as an open system, consisting of a variable number of members, living in the same place, legally united, by blood or affinity.’ Family functionality (FF) involves the interaction of various integrators and modifications of family bonds, which may lead to alterations.

According to the FF-SIL families can be classified into different levels of functionality: First, FF, characterised by cohesive relationships, effective communication and appropriate conflict resolution. This can contribute to a healthy family environment. Second, moderately FF, which shows some difficulties in interaction or adaptation but maintains the ability to respond effectively to crises. Third, a dysfunctional family, which is distinguished by disorganisation, poor communication and a lack of mutual support among members. Finally, a severely dysfunctional family exhibits a serious deterioration of family bonds and structure, which significantly affects the physical and emotional well-being of its members.

Coping is defined as ‘a dynamic, adaptive cognitive behavioural effort to meet internal or external demands, perceived as excessively demanding of individual resources.’

There are two types: Primary (problem-focused), which is oriented towards action and the resolution of the stressful situation and Secondary (emotion-focused), which is aimed at regulating affective responses. Each of these dimensions includes various subscales that reflect adaptive or maladaptive strategies, such as seeking social support (SS), positive reappraisal, avoidance or self-criticism (SC).

Therefore, understanding both constructs allows for the analysis of how family dynamics influence the choice of coping strategies and, consequently, the psychological well-being of individuals.

To date, no longitudinal studies have evaluated the evolution of FF and coping strategies in patients with BC and T2DM. Therefore, the objective of this study was to evaluate the evolution of FF and coping in patients with strategies in and T2DM.

## Method

This longitudinal cohort study was conducted in the Mexican Institute of Social Security in Cancun Quintana Roo from 2019 to 2023.

Patients diagnosed with BC and a prior diagnosis of T2DM were included after providing informed consent and agreeing to follow-up. Patients with comorbidities affecting other organs who incorrectly completed the questionnaires, those who did not sign the informed consent or those who withdrew from the study were excluded. This study included patients with a recent cancer diagnosis who agreed to participate and completed a 4-year follow-up. Patients who failed to complete the 4-year follow-up were excluded.

The primary variable of interest was FF and baseline coping at the time of the initial BC diagnosis and after 4 years of progression. FF was assessed using the FF-SIL test, designed to evaluate intrafamilial relationships with a temporary reliability of 0.93 and high internal consistency (*α* = 0.85). The questionnaire includes 14 questions, with responses categorised as: Almost never, Rarely, Sometimes, Often and Almost always. Scores were interpreted as follows: 57–70 indicates FF; 43–56, moderately FF; 28–42, dysfunctional family and 14–27, severely dysfunctional family.

Coping was measured using the coping level (CSI) test, which demonstrates internal consistency levels ranging from 0.63 to 0.80. This tool comprises of 40 questions with responses rated as follows: not at all, slightly, moderately, very much and completely. The survey was completed within 15–20 minutes, with opportunities provided to address any questions from participants.

Epidemiological data were collected through a questionnaire, medical records and laboratory tests (fasting glucose, glycated haemoglobin, cholesterol and triglycerides). Additional data included age, occupation, marital status, education level, cancer diagnosis and treatment, DM2 treatment, current weight and 1-year follow-up records from family medicine and oncology at Regional General Hospital No. 17.

The collected data were entered into a statistical database. Descriptive statistics were then used, including measures of central tendency and dispersion (range, mean, median, mode, standard deviation, proportions or percentages). Inferential statistics were applied due to the presence of two or more samples. The Wilcoxon test was used for related samples with a significance level set at 0.05.

Ethical research principles were followed according to the Declaration of Helsinki and its Tokyo amendments for biotic research, adhering to the guidelines for health research.

This project was reviewed and approved by the local ethics committee, registered under Conbioética 23 CEI 001 2017103, the local health research committee registration 2301, institutional registration number R-2022-2301-009 and COFEPRIS registration 17 CI 23 005 128.

## Results

Only 18 patients completed the 4-year cohort period; there were 47 patients who were excluded due to loss of social security. In 2019, 21 patients had an adequate/dysfunctional coping strategy, 34 had an inadequate/dysfunctional coping strategy, five had adequate/functional and 5 had an inadequate/functional coping strategy. Compared to 2023, which found that 3 patients had an adequate/dysfunctional coping strategy, 5 had inadequate/dysfunctional, 6 had adequate/functional and 4 had an inadequate/functional coping strategy (Table 1).

FF with FF-SIL in 2019 presented a median of 51.10 (48.83,53.37) versus the FF-SIL test of 2023, which had a median of 44.72 (39.19,50.25); the difference between medians was 5.7 *p* = 0.003 (Table 2).

FF was considered in four sections: 1) FF 27.7% (18,40.4) versus 27.8% (5.6,50); 2) Moderately dysfunctional 56.9% (44. 2, 68.1) versus 16.7% (4,34.9); 3) Dysfunctional 12.3% (6.2, 23.5) versus 55.6% (33.3,79.4); 4) Severely dysfunctional 3.1% (0,7.7) versus 0 with *p*-value = 0.42 (Table 3).

Coping by CSI test shows eight variables: 1) Problem solving (PS) 4.6% (0.10.8) versus 6.2% (5.6,44.4), 2) SC 24.6% (14.9,33.8) versus 4.6% (0.34.9), 3) Emotional expression (EE) 21.5% (11,32.3) versus 4.6% (0,38.9), 4) Desiderative thinking (DT) 26.2% (14.9,37.4) versus 1.5% (0,18. 3), 5) SS 4.6% (0.11) versus 1.5% (0.18.3), 6) Cognitive restructuring (CRE) 3.1% (0.9.7) versus 4.6% (0.38.9), 7) Problem avoidance (PAV) 6.2% (1. 5,13.8) versus 3.1% (0,27.8) and 8) Social withdrawal (SW) 9.2% (1.5,20.4) versus 1.5% (0,22.2) with 95% significance and *p* = 0.59 not significant (Table 4).

The results of those who were monitored by the Family Medicine Unit found that 56% were not monitored, 44% went for a medical consultation; of this percentage, 50% went for 4 annual consultations. 78% did not have any type of psychological follow-up, 22% went for a mental health check-up; of this group, 75% had psychological therapy and 25% psychiatric therapy (Table 5).

## Discussion

In Mexico in 2022, the incidence of BC was 23,790 cases among individuals aged 20 years and older, equating to 27.64 cases per 100,000 individuals. BC accounted for 9% of deaths caused by malignant tumours (7,888 cases), with a mortality rate of 11.26 deaths per 100,000 women aged 20 years and older. In Quintana Roo, the rate ranged from 37.95 to 55.27 per 100,000 individuals. After the age of 35, the risk of death among BC patients increased, peaking at 51.3% among those older than 60. The state’s BC index has risen by 63% since 2018.

Limited incidence and mortality records in the southern region of the country have been associated with geographical and cultural barriers in marginalised areas, hindering the timely detection of neoplastic diseases. In our sample, the average age at diagnosis was 59.32 years, with 55.4% of the population being younger than 60 years, highlighting a greater incidence and age-related mortality risk.

Modifiable risk factors, including being overweight, obesity, dyslipidaemia and physical inactivity, remain prevalent. Women aged 20–69 years old report an average of less than 2 hours of weekly physical activity.

At diagnosis, the average body mass index (BMI) in this study was 29.83 kg/m², indicating obesity. Over 4 years, there was a reduction of 2.62 kg/m² in BMI, likely due to the pathological condition faced by the women. Nevertheless, the presence of obesity was already evident in this sample.

Non-modifiable factors include a personal, family history of BC and genetic mutations, particularly in the BRCA1 and BRCA2 genes. Between 2003 and 2008, most diagnoses (70.2%) were at advanced stages with a high mortality risk. The national consensus on institutional screening in Mexico recommends starting at age 40, resulting in a mortality reduction of 40% for patients aged 50%–69% and 29–48% for those aged 40–49, thanks to timely detection programmes and mammography use.

In this study, more than 50% of the population received early diagnosis based on age, indirectly reflecting the impact of early detection programmes. However, the remaining 44.7%, aged over 60, highlight a need to investigate the causes of delayed detection.

In 2019, León R. reported that 50.45% of BC patients had a family history, 28.44% had concurrent BC and T2DM and 44.03% had hypertension. Approximately 32.2% developed comorbidities. Sites of cancer strongly associated with type 2 diabetes, as indicated by a 2021 meta-analysis, include colorectal, breast, endometrial, gallbladder, hepatocellular and pancreatic cancers, which are also linked to being overweight and obese. Previous studies estimate that more than 800,000 new cancer cases annually are attributable to the combination of high BMI and diabetes, increasing the risk for six specific cancer types. Although this association was not observed in our study, all patients had DM2 and 78.5% were overweight or obese.

Psychological distress was clinically relevant in 40% of the patients, with symptoms such as depression (0%–58%), anxiety (6%–23%) and post-traumatic stress disorder (0%–32%). BC is a significant stressor for physical and psychological well-being. Comprehensive evaluations revealed that 83.3% of patients relied on emotional coping, 68% on self-control, 20% on positive re-evaluation and 12% on social distancing. These factors impact the emotional states and overall FF.

Only 7.6% of patients initially exhibited both adequate FF and coping strategies. One notable finding was the progression in coping mechanisms for 33% of patients and FF (*p* = 0.5), which was clinically significant but not statistically significant due to cohort attrition.

Educational level significantly affects the management of T2DM and BC, with higher education levels enabling a better understanding of the pathology and treatment. Family context and SS are crucial for managing these diseases, underscoring the importance of tailored interventions that address individual and familial needs.

In 2023, a greater proportion of patients lacked a regular follow-up compared to 2019, indicating challenges in healthcare management. These are attributed not to healthcare institutions but to patient responsibility, empowerment and socioeconomic barriers to self-care.

Most patients did not receive mental health support despite the psychological burden of their diagnoses. Differences in follow-up revealed notable changes in disease progression and therapeutic impact. Longer durations of chronic illness were associated with reduced optimism and less effective coping strategies, highlighting the emotional and psychological toll.

Negative coping styles, such as resignation, protest or isolation, are common in women with severe illnesses, leading to a poorer quality of life. Educational and mental health interventions can promote therapeutic adherence and overall health, as evidenced by this study’s findings on coping and family dynamics.

Family typology influences disease management, psychological well-being and emotional health, emphasising the need for a holistic biopsychosocial approach. Extended families played a greater supportive role, with children often serving as primary caregivers.

Over time, a decline in FF and effective coping strategies was observed, reflecting accumulated stress. While some families adapted, others experienced a deterioration in dynamics, underscoring the need for targeted psychosocial interventions.

## Limitations

The study faced significant attrition, which may limit the generalisability of the findings. Additionally, several statistical tests did not achieve significance, suggesting the need for larger sample sizes or alternative methodologies.

## Conclusion

This study contributes to understanding the evolution of FF and coping strategies in patients with BC and T2DM, highlighting the importance of early and ongoing psychosocial interventions. Chronic disease management must incorporate effective and adaptive coping strategies to improve outcomes, particularly in older populations.

## Conflicts of interest

The authors report there are no competing interests to declare.

## Funding

This work was supported by a Grant from the Mexican Institute of Social Security Research Committee registration 2301 with institutional registration number R-2022-2301-009, and COFEPRIS registration 17 CI 23 005 128.

## Author contributions

María Valeria Jiménez-Báez

Project administration, Methodology, Formal analysis, Investigation, Writing - Original Draft, Supervision.

Sofía Concepción Thomas-Gómez

Writing - Original Draft, Data Curation, Conceptualisation, Software.

Gabriel González-Guerrero

Writing - Review & Editing, Visualization, Data Curation.

David Rojano-Mejía

Validación, Investigation, Data Curation.

Eduardo Patricio Achurra-Godinez

Writing - Review & Editing, Visualisation, Data Curation.

## Figures and Tables

**Table 1.** Differences between FF SIL and CSI 2019–2023.

		2019			2023		
		(n = 65)			(n = 18)		
		Adequate	Inadequate	*p* = 0.3	Adequate	Inadequate	*p* = 0.5
FF SIL	Dysfunctional	21 (32.3%)	34 (52.3%)		3 (16.6%)	5 (27.7%)	
	functional	5 (7.6%)	5 (7.61%)		6 (33.3%)	4 (22.2%)	

Chi square *χ*2

**Table 2.** Differences 2019–2023.

Differences CSI 2019–2023				
	*N*	Mediana	RIC (25)	RIC (75)
Results CSI 2019	65	4	2.00	4.50
Results CSI 2023	18	3	2.00	6.00
Wilcoxon *p*-value *p* = 0.006				
Differences FF SIL 2019–2023				
Score FF SIL 2019	65	52	47	36
Score FF SIL 2023	18	40	59	58
Wilcoxon* p*-value *p* = 0.003				

**Table 3.** FF.

	2019				2023				
	N = 65	%	95% Confidence interval		N = 18	%	95% Confidence interval		p
			Inferior	Superior			Inferior	Superior	
Functional	18	27.7	18	40.4	5	27.8	5.6	50	0.42
Moderately dysfunctional	37	56.9	44.2	68.1	3	16.7	4	34.9	
Dysfunctional	8	12.3	6.2	23.5	10	55.6	33.3	79.4	
Severely dysfunctional	2	3.1	0.0	7.7					

CI 95% Frequency (*N*), Percentage (%), *p*-value (*p*), Chi square *χ*^2^

**Table 4.** CSI.

	2019				2023				*p*
	*N* = 65	%	95% Confidence interval		*N* =18	%	95% Confidence interval		
			Inferior	Superior			Inferior	Superior	
PS	3	4.6	0	10.8	4	6.2	5.6	44	0.59
SC	16	24.6	14.9	33.8	3	4.6	0	34.9	
EE	14	21.5	11	32.3	3	4.6	0	38.9	
DT	17	26.2	14.9	37.4	1	1.5	0	18.3	
SS	3	4.6	0	11	1	1.5	0	18.3	
CRE	2	3.1	0	9.7	3	4.6	0	38.9	
PAV	4	6.2	1.5	13.8	2	3.1	0	27.8	
SW	6	9.2	1.5	20.4	1	1.5	0	22.2	

CI 95% Frequency (*N*), Percentage (%), *p*-value (*p*) Chi square *χ*^2^

**Table 5.** Sociodemographic characteristics.

Marital status	
Married Widow Single	7 (39%) 1 (5%) 10 (56%)
Visits in UMF*	8 (44%)
Number of visits UMF*	
0 1 2 3 4	10 (56%) 1 (5%) 2 (11%) 1 (1%) 2 (17%)
Oncological control	12 (66.6%)
Therapy	4 (22%)
Type of therapy	
Psychological Psychiatric	3 (75%) 1 (25%)
Primary caregiver	
Partner Offspring By itself	3 (16.5%) 9 (50%) 6 (33.5%)
Primary authority	
Partner Offspring By itself	4 (22.2%) 3 (15.5% 11 (61.2%)

Frequency (*n*), Percentage (%), *UMF First Contact Family Medicine Unit

